# Development of a dedicated phantom for multi‐target single‐isocentre stereotactic radiosurgery end to end testing

**DOI:** 10.1002/acm2.12452

**Published:** 2018-09-16

**Authors:** Joel Poder, Ryan Brown, Harry Porter, Rashmi Gupta, Anna Ralston

**Affiliations:** ^1^ St George Hospital Cancer Care Centre Kogarah NSW Australia

**Keywords:** dosimetric validation, end‐to‐end testing, MRI deformation, multi‐target, single isocentre, stereotactic radiosurgery

## Abstract

**Purpose:**

The aim of this project was to design and manufacture a cost‐effective end‐to‐end (E2E) phantom for quantifying the geometric and dosimetric accuracy of a linear accelerator based, multi‐target single‐isocenter (MTSI) frameless stereotactic radiosurgery (SRS) technique.

**Method:**

A perspex Multi‐Plug device from a Sun Nuclear ArcCheck phantom (Sun Nuclear, Melbourne, FL) was enhanced to make it more applicable for MTSI SRS E2E testing. The following steps in the SRS chain were then analysed using the phantom: magnetic resonance imaging (MRI) distortion, planning computed tomography (CT) scan and MRI image registration accuracy, phantom setup accuracy using CBCT, dosimetric accuracy using ion chamber, planar film dose measurements and coincidence of linear accelerator mega‐voltage (MV), and kilo‐voltage (kV) isocenters using Winston‐Lutz testing (WLT).

**Results:**

The dedicated E2E phantom was able to successfully quantify the geometric and dosimetric accuracy of the MTSI SRS technique. MRI distortions were less than 0.5 mm, or half a voxel size. The average MRI‐CT registration accuracy was 0.15 mm (±0.31 mm), 0.20 mm (±0.16 mm), and 0.39 mm (±0.11 mm) in the superior/inferior, left/right and, anterior/posterior directions, respectively. The phantom setup accuracy using CBCT was better than 0.2 mm and 0.1°. Point dose measurements were within 5% of the treatment planning system predicted dose. The comparison of planar film doses to the planning system dose distributions, performed using gamma analysis, resulted in pass rates greater than 97% for 3%/1 mm gamma criteria. Finally, off‐axis WLT showed MV/kV coincidence to be within 1 mm for off‐axis distances up to 60 mm.

**Conclusion:**

A novel, versatile and cost‐effective phantom for comprehensive E2E testing of MTSI SRS treatments was developed, incorporating multiple detector types and fiducial markers. The phantom is capable of quantifying the accuracy of each step in the MTSI SRS planning and treatment process.

## 1 INTRODUCTION

Intracranial metastases are being discovered in approximately 400,000 patients per year worldwide,^1^ of which about 70–80% will have multiple intracranial metastases.^2^ The role of linear accelerator based stereotactic radiosurgery (SRS) in treatment of these intracranial metastases has expanded significantly in past decades, due to its high delivery efficiency^3^ and equivalent plan quality,^4^ relative to Gamma Knife (GK) radiosurgery.

Recent published data indicates that due to the relatively high toxicity and poor neurological outcomes associated with whole brain radiotherapy, SRS is now becoming the standard of care in the treatment of patients with multiple brain metastases.^5^, ^6^, ^7^ The role of SRS has recently expanded to include treatment of multiple cranial metastases with a single isocenter,^8^, ^9^ hence further reducing treatment times. This technique requires the use of multi‐leaf collimators (MLCs) and volumetric modulated arc therapy (VMAT) or dynamic conformal arc therapy (DCAT), as cone collimation can treat only one target at a time.

Another recent change in technique has been the use of on‐board imaging, such as cone beam computed tomography (CBCT) facility on the linear accelerator. Rather than relying on a separate kV X‐ray imaging system, CBCT is used to position the patient for SRS. This requires much tighter tolerances for the linear accelerator isocenter and CBCT geometric accuracy compared to non‐SRS treatments.

To meet the growing demand, there is a need for SRS treatments to be available in most radiation oncology departments rather than remain confined to specialist centers. This creates a challenge for staff in departments who do not have SRS experience or the appropriate equipment, to meet the strict demands on geometric and dosimetric accuracies required for the multi‐target single‐isocenter (MTSI) SRS technique.

An essential element of setting up and maintaining an SRS program is quantifying the uncertainties inherent in the planning and treatment process. These factors include, but are not limited to, uncertainties due to systematic, and/or random errors in: gross tumour volume (GTV) contouring,^10^ geometric distortion of magnetic resonance imaging (MRI) used for contouring of GTVs,^11^ the image registration of MRI and CT images used for treatment planning,^12^ the measurement of small field data,^13^ the treatment planning system (TPS) modeling of small field sizes,^14^ differences in the treatment and imaging isocenters of the linear accelerator,^15^ patient positioning after CBCT,^15^ and intra‐fraction motion of the patient during treatment delivery.^16^

The aim of end to end (E2E) testing is to measure the overall geometric and dosimetric accuracy of the planning and treatment chain. Several previously published studies have successfully achieved this for SRS,^10^, ^17^, ^18^, ^19^ however, none of these quantified the geometric and dosimetric accuracy for MTSI SRS. The characteristics of an ideal E2E phantom for MTSI SRS include:

- Compatibility with MRI, CT, CBCT and MV imaging
- Dimensions and shape similar to an average human head
- Cost effectiveness
- External markings for quick and easy setup
- Compatibility with patient immobilization equipment
- No dose perturbation for non‐coplanar fields (i.e., non‐zero couch angles)
- Minimal air gaps
- Ability to position fiducial markers, point detectors, and radiochromic film over a wide range of locations, including those close to the lateral and superior edges of the phantom in order to encompass off‐axis targets

There are several commercially available devices that are marketed for MTSI SRS quality assurance (QA), such as: the Sun Nuclear SRS MapCheck, Standard Imaging Lucy 3D, Integrated Medical Technologies MAX‐HD, and CIRS Steev phantoms. However, these phantoms may not meet all the criteria listed above or may have a cost that is prohibitive to a department in the initial stages of developing an SRS program. The design, manufacture, and use of a low‐cost, versatile, MTSI SRS E2E phantom that meets all the criteria listed above, through the enhancement of a Multi‐Plug device from a Sun Nuclear ArcCheck phantom (Sun Nuclear, Melbourne, FL) is presented.

## 2 MATERIALS AND METHODS

### 2.A Phantom design

**Figure 1:**
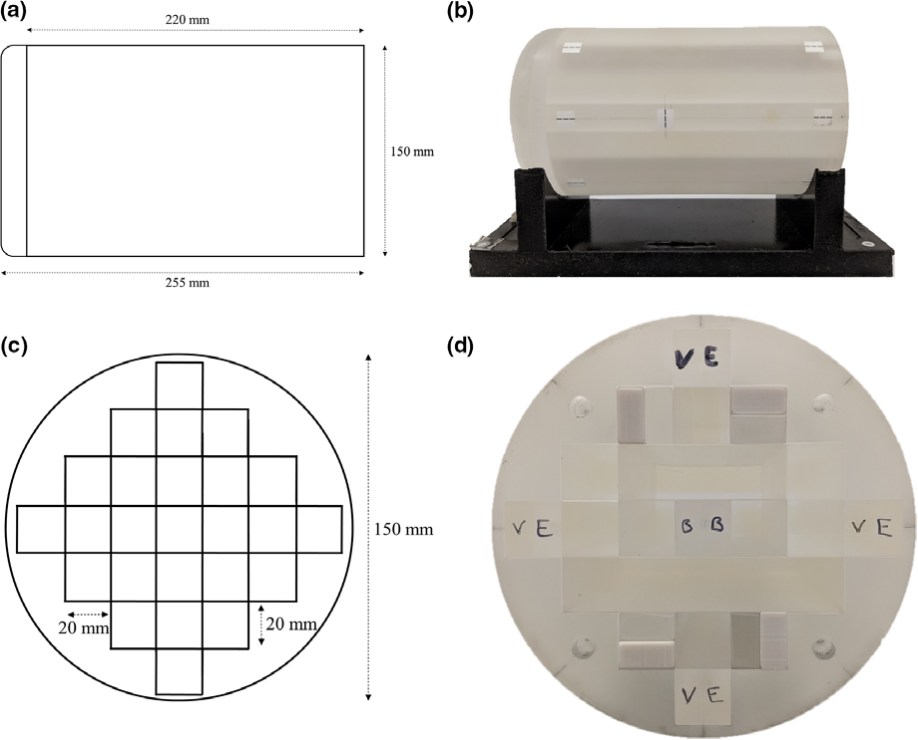
(a) Side‐on schematic of MTSI SRS E2E phantom, (b) Side‐on photograph of the MTSI SRS E2E phantom, (c) End‐on schematic of MTSI SRS E2E phantom, (d) End‐on photograph of the MTSI SRS E2E phantom.

The phantom is a Perspex cylinder of diameter 150 mm and length 255 mm. The central portion of the phantom is made up of Perspex inserts (20 × 20 × 220 mm) to allow for placement of dosimeters and fiducial markers, to aid in the SRS E2E testing process (Fig. 1). The size and shape of the MTSI phantom without the ArcCheck is ideal for MTSI SRS E2E testing as it closely mimics the size and shape of an average adult head and allows for a thermoplastic mask to be fitted to the phantom to provide a true E2E test incorporating all relevant equipment. Minor modifications were made to the phantom, e.g., removal of the metal handle and threaded inserts to avoid MRI distortion, and rounding of the cylinder end through which the vertex fields enter (note that this rounding does not affect the use of the MultiPlug in the ArcCheck). Additionally, external markings were added to the surface of the phantom to ensure reproducible setup of the phantom within the thermoplastic mask in the time between simulation and treatment. The coordinate system for the phantom was chosen to be the same as that for a supine patient, i.e., superior/inferior (toward/away from the gantry for couch 0°), right/left (patient right/left) and anterior/posterior (up/down).

**Figure 2:**
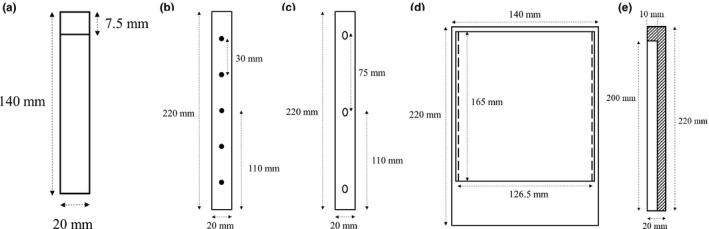
(a) Phantom insert for PTW PinPoint3D ionization chamber, (b) ball bearing insert, (c) Vitamin E insert, (d) GafChromic EBT3 film insert, (e) bone analogue insert.

Two of the inserts used for SRS E2E testing in this study were supplied by Sun Nuclear: an insert for a PTW PinPoint3D Type 31016 ionization chamber [Fig. 2(a)] and an insert for a 126.5 × 165 mm piece of radiochromic film [Fig. 2(d)]. The PTW PinPoint3D chamber insert was modified by cutting its length to 140 mm, so that the end of the insert was 7.5 mm beyond the center of the chamber sensitive volume, and a range of short Perspex lengths (5, 10, 20, 30, and 50 mm) were manufactured to allow placement of the effective point of measurement of the chamber at various positions along the length of the phantom. The radiochromic film insert was modified by narrowing the film cavity [from solid lines to dashed lines in Fig. 2(d)] so that its width was exactly equal to half the width of a piece of Gafchromic EBT3 film. This allows for less wastage of film, as one piece of Gafchromic EBT3 film can be used for two phantom measurements and one set of calibration measurements. Additional Perspex inserts were made which were full length but with a reduced cross section of 5 × 20 mm or 10 × 20 mm. These additional Perspex components enable detector placement points within the phantom with 5 mm increments in every direction (superior/inferior, left/right, and anterior/posterior).

Other inserts used for the E2E testing were made in‐house: five Perspex inserts, each implanted with three vitamin E capsules for MRI distortion testing [Fig. 2(c)], one Perspex insert with five steel ball bearings (BB) for performing on‐ and off‐axis Winston‐Lutz tests (WLT) [Fig. 2(b)], and four L‐shaped inserts made from a commercially available bone‐equivalent material [Fig. 2(e)] used for CBCT positioning. The vitamin E capsules had length 13 mm and width 6 mm, and the BB diameter was 5 mm. Each vitamin E and BB insert was made by creating appropriately sized hollows in a pair of 10 × 20 × 220 mm inserts, placing the vitamin E capsules or BBs in the hollows, and joining the two halves with Perspex compatible glue. Vitamin E capsules were chosen as they are readily available, low cost and have been shown to have excellent contrast on both MRI and CT scans.^11^ The bone‐equivalent inserts were designed so that positioning of the phantom in the superior/inferior direction could be optimized using the additional information provided by the L‐shape. The bone‐equivalent material had a cross section of 10 × 20 mm and the rest of the insert (total cross section 20 × 20 mm) was filled in with Perspex.

The use of the phantom for SRS E2E testing involves minimal cost for departments who have already purchased an ArcCheck with Multi‐Plug, with the only additional cost being that of constructing the custom inserts (approximately $300 in parts and labour). Alternatively, the Multi‐Plug insert may be purchased alone, significantly reducing the initial cost compared to purchasing the full ArcCheck device or a dedicated SRS head phantom. If there is no facility for manufacturing the custom inserts in‐house, these can be outsourced to a plastic cutting firm, preferably with laser cutting capability. If the inserts are initially too tight to slide in and out easily they can be incrementally sanded back to get an ideal fit without introducing significant air gaps. In our study the bone equivalent inserts were made from a larger slab of this material, however, bone equivalent inserts for the Multiplug can be purchased from Sun Nuclear. Alternatively, another medium density material (approximately 2–3 g/cm^3^) could be used if it was able to be laser cut or machined and did not introduce artefacts in MRI or CT scans. The advantage of the L‐shaped bone inserts used in this study [Fig. 2(e)] is that improved positioning accuracy in the superior/inferior direction could be achieved, relative to those that can be purchased from Sun Nuclear.

### 2.B Treatment planning approach

#### 2.B.1 Imaging

The phantom was initially scanned using a 3T Philips Achieva MRI scanner with a T1 weighted, 1 mm slice thickness MRI scan protocol. To provide fiducial markers for assessment of MRI distortion and MRI/CT image fusion accuracy, the five vitamin E inserts [shown in Fig. 2(c)] were placed in the center, most lateral extents, and most anterior/posterior extents of the phantom.

From the MRI scan, the distances between the centroids of each vitamin E capsule were measured in the treatment planning system and compared to the known distances (within the mechincal uncertainty of ±0.2 mm) to quantify the effects of any image distortion across the range of the entire image. The average, standard deviation, and root mean squared (RMS) errors were calculated for each direction independently.

**Figure 3:**
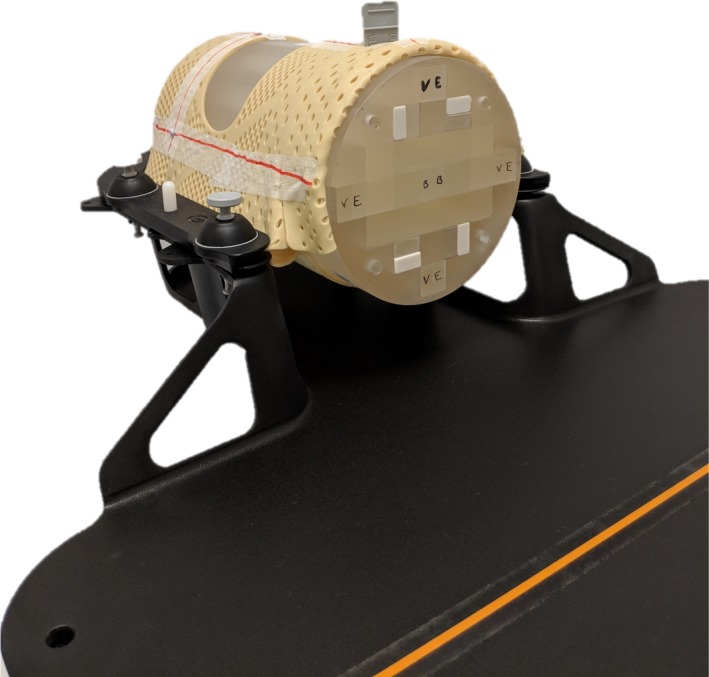
MTSI SRS E2E phantom placed inside the QFix Encompass SRS headboard and mask system.

**Figure 4:**
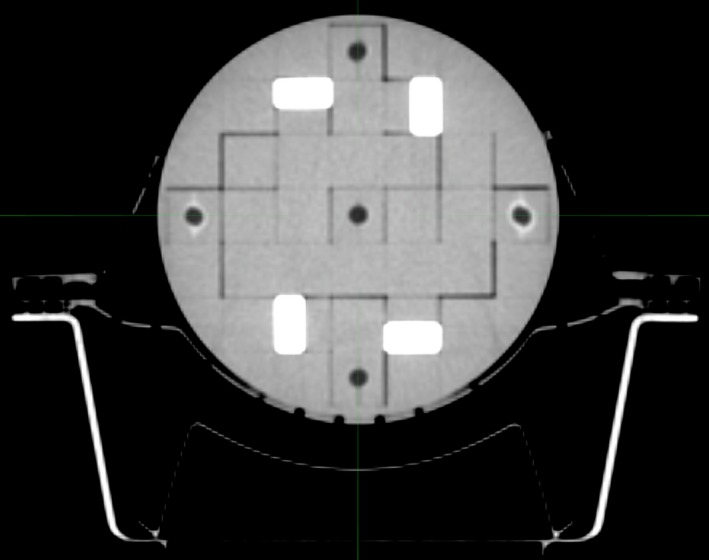
Axial CT slice indicating orientation of bone inserts in E2E phantom.

The phantom was placed within a QFix Encompass SRS headboard (Type RT‐4600‐01, QFix, Avondale, PA) and mask system (Type RT‐B889KYCF2, QFix, Avondale, PA), the same system to be used for patient localization during the SRS treatment planning and delivery process in our department (Fig. 3). The phantom was then scanned with 1 mm slice thickness on a dedicated radiotherapy Philips Big Bore CT scanner with the vitamin E inserts in the same configuration as the MRI, along with four bone inserts as shown in Fig. 4. The setup and CT scan of the phantom, as well as construction of the mask was performed by radiation therapists, to mimic the true patient simulation as closely as possible. The scan was exported to the image registration software platform.

#### 2.B.2 Registration MRI‐CT

**Figure 5:**
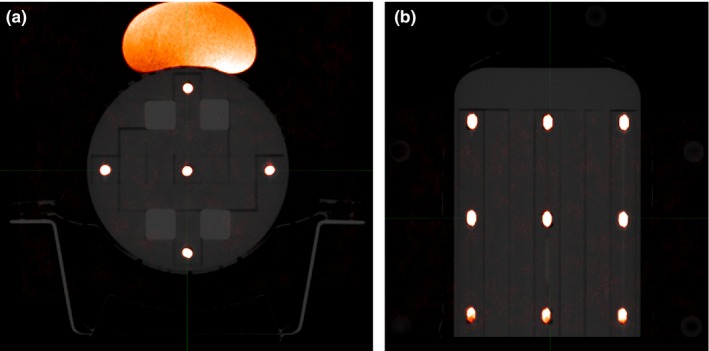
MRI/CT fusion of E2E phantom in (a) axial, and (b) coronal orientations.

The MRI scan was registered to the CT using a manual rigid registration in the MIM (v6.7.10, MIM Software Inc., Cleveland, OH) imaging platform. The registration was performed by a radiation therapist and independently reviewed by a physicist and radiation oncologist. The image registration was based on alignment of the vitamin E capsules in the images, as shown in Fig. 5. To calculate the image registration uncertainty, three different radiation therapists performed the registration retrospectively. The deviation between the centroid of the vitamin E capsule on the MRI and CT images was calculated for each capsule in each direction (superior/inferior, left/right, anterior/posterior) for each of the three registrations. Finally, the average, standard deviation, and RMS error of the deviations were calculated for each direction.

#### 2.B.3 Treatment plans

To perform the dosimetric validation of the technique, several MTSI SRS treatment plans were created on the CT scan using the Pinnacle TPS (v9.10, Philips, Fitchburg, WI). A total of 10 SRS plans were created on the CT scan consisting of 1–4 spherical isocentric or non‐isocentric target volumes (Table 1). A volumetric modulated arc therapy (VMAT) plan technique was used within the SmartArc module of Pinnacle with the collapsed‐cone convolution algorithm^19^ to create the plans, following the University of Alabama MTSI SRS treatment planning protocol that has been described previously.^4^ The plans were created with 5 mm width multi‐leaf collimators (Varian Millenium 120 leaf MLC) on a TrueBeam (v2.5, Varian Medical Systems, Palo Alto, CA) linear accelerator with a 6 MV flattening filter free (6FFF) beam energy.

**Table 1:** Summary of SRS plans used for E2E validation

Plan	Number of targets	Target diameter (mm)	Off‐axis distances (mm)
1	1	10	0
2	1	20	0
3	1	30	0
4	1	40	0
5	2	10, 20	30 (sup), 30 (inf)
6	2	10, 30	30 (left), 30 (right)
7	2	20, 40	60 (ant), 60 (post)
8	2	10, 20	60 (sup), 60 (inf)
9	4	10, 20, 30, 40	30 (sup), 30 (inf), 30 (left), 30 (right)
10	4	10, 20, 30, 40	60 (ant), 60 (post), 60 (left), 60 (right)

#### 2.B.4 Phantom setup and image guidance

For each plan, the phantom was placed in the dedicated SRS headboard and mask system by the radiation therapists and initially aligned using the lasers and the reference marks that were placed on the mask system at CT simulation. A CBCT was then performed and the phantom aligned to the reference CT scan using a bone window within the Varian TrueBeam On Board Imaging software (Varian Medical Systems, Palo Alto, CA) and a 6 degree of freedom (6DOF) Perfect Pitch couch. The bone inserts placed within the phantom were used to perform the CBCT match. The use of the bone window and inserts mimics closely the true clinical scenario in which the skull of the patient is used for CBCT matching. A repeated CBCT scan was then acquired to test the reproducibility of the setup, as indicated by a zero offset required after the second CBCT scan.

#### 2.B.5 Off‐axis WLT

Prior to the CBCT match, the ball bearing (BB) insert was placed in the phantom at so that a BB was aligned to the target location as defined by the treatment plan described in Section 2.B.3. A multi‐leaf collimator (MLC) defined 2 × 2 cm field size was created with the collimator jaws set to 3 × 3 cm, centerd on the target in the TPS. After the CBCT match, the MLC field was delivered and image captured on an electronic portal imaging device (EPID) for each combination of gantry, collimator, and couch angles outlined in Table 2. The BB position relative to the field center was then calculated using the DoseLabPro software package (v6.40, Mobius Medical Systems, Redwood City, CA). This procedure was repeated for each of the targets in the treatment plans.

**Table 2:** Combination of gantry, collimator, and couch fields used for Winston‐Lutz testing in this study

Gantry angle (°)	Collimator angle (°)	Couch angle (°)
180	0	0
270	0	0
0	0	0
0	90	0
0	270	0
90	0	0
0	0	90
0	0	270

#### 2.B.6 Point dose measurements

Following the CBCT setup and off‐axis WLT, point dose measurements were performed on the treatment plan with a PTW PinPoint3D ionization chamber with a dedicated insert [Fig. 2(a)]. The center of the chamber sensitive volume was placed at the center of the target as defined in the TPS. A region of interest was created in the TPS with identical volume to that of the PTW PinPoint3D ionization chamber, centerd on the point of interest. The average computed dose in this volume was compared to the measured point doses with a tolerance of 5%.

#### 2.B.7 Planar dose measurements

2D planar dose measurements were performed using Gafchromic EBT3 film (Ashland Inc., Wayne, NJ, USA) of size 125 × 165 mm. Planar doses were measured through the center of each target and compared to those predicted by the TPS, using gamma analysis.^20^ A tolerance of 3% global dose difference and 1 mm distance to agreement was employed (excluding dose points below a 10% threshold) with a minimum pass rate of 95% considered acceptable.

EBT3 films were scanned in an Epson 10000XL flatbed scanner (SEIKO Epson Co, Japan). The films were positioned on the scanner with a jig to ensure reproducible positioning of the film within the scanning area, and placed under a sheet of glass during scanning to maintain good contact between the film and the scanner as recommended by Ashland.^21^ The films were scanned 24 h after exposure to allow stabilization of the latent image.^22^

Each film was scanned in transmission mode using 48 bit RGB with a scanner resolution of 75 dpi (0.34 mm pixel size). All phantom and calibration film pieces were marked in order to maintain the same orientation during scanning, thereby eliminating polarization effects,^23^ and positioned at the center of the flatbed scanner with the long axis of the film pieces parallel to the long axis of the scanner to avoid off‐axis scanner non‐uniformity.^24^ The pixel values measured from the red channel of the scanner were used to calculate the optical density (OD) for each film piece and these were converted to absorbed dose (in cGy) using a OD‐to‐dose calibration curve.

## 3 RESULTS

### 3.A MRI distortion

The average (±1 SD) distortion was measured in MIM as 0.15 mm (±0.31 mm), 0.20 mm (±0.16 mm), and 0.39 mm (±0.11 mm) in the superior/inferior, left/right, and anterior/posterior directions, respectively. The RMS distortion was 0.32, 0.23, and 0.45 mm in the superior/inferior, left/right, and anterior/posterior directions, respectively. The maximum difference between known and MRI measured distances was less than 0.5 mm, or half a voxel size. The maximum difference between the known and MRI measured distances was comparable to the known mechanical uncertainty (±0.2 mm) of embedding the vitamin E capsules within their respective inserts.

### 3.B MRI/CT fusion

The average (±1 SD) MRI/CT fusion error was measured in MIM as 0.23 mm (±0.12 mm), 0.19 mm (±0.25 mm), and 0.29 mm (±0.12 mm) in the superior/inferior, left/right, and anterior/posterior directions, respectively. The RMS fusion error was 0.19, 0.22, and 0.29 mm in the superior/inferior, left/right, and anterior/posterior directions, respectively. For each capsule, the fusion errors in each direction were measured to be less than 0.5 mm, or half a voxel size.

### 3.C. Phantom setup and CBCT alignment

Phantom setup and CBCT alignment results are summarized in Tables 3 and 4, for translations and rotations, respectively. As can be seen from the tables, after CBCT based correction using a 6DOF couch, the residual translation and rotation errors after repeat CBCT are 0.2 mm and 0.1°, respectively. Furthermore, the relatively minor initial corrections indicate that a reproducible setup of the phantom between simulation and treatment.

**Table 3:** Initial and residual phantom translational alignment using CBCT and a 6DOF couch

	Initial (mm)	Residual (mm)
Longitudinal	1.2	0.2
Lateral	0.9	0.1
Vertical	0.9	0.2

**Table 4:** Initial and residual phantom rotational alignment using CBCT and a 6DOF couch

	Initial (mm/°)	Residual (mm/°)
Pitch	0.3	0.1
Roll	0.4	0.0
Yaw	0.0	0.0

### 3.D Off‐axis WLT

**Figure 6:**
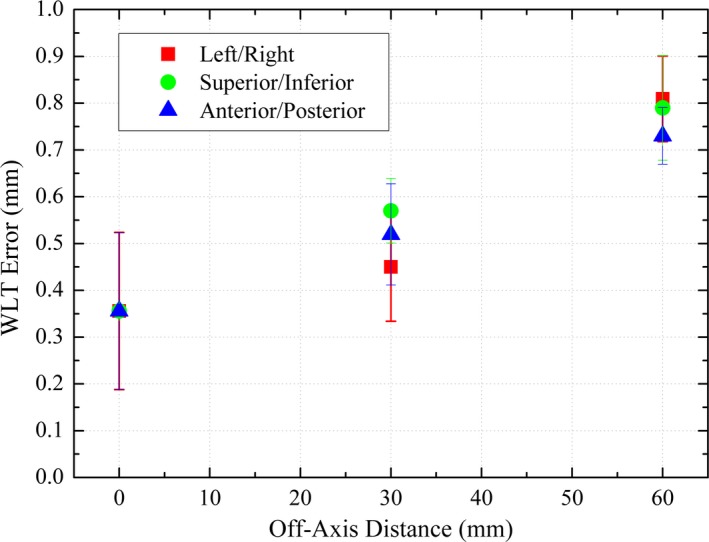
Average Winston‐Lutz test result (±1 SD), plotted as a function of distance away from isocenter for each of the three major axes (left/right, superior/inferior, anterior/posterior).

The off‐axis WLT results are summarized in Fig. 6 and Table 5, where the average displacement from the center of the BB to the center of the radiation field is shown for each off‐axis distance (±1 SD) for each of the three major axes (left/right, superior/inferior, anterior/posterior). As the off‐axis distance increased, there was an observed trend of increasing displacement between the center of the BB and the center of the radiation field. However, even for an off‐axis distance of 6 cm, the maximum deviation was 0.97 mm.

**Table 5:** Summary of the displacement of the center of the ball bearing from the center of the radiation field, as a function of off‐axis distance from the MV isocenter

Off axis distance (mm)	Average (±1 SD) displacement (mm)	RMS displacement (mm)	Max. displacement (mm)
0	0.36 ± 0.17	0.51	0.72
30	0.51 ± 0.10	0.49	0.88
60	0.76 ± 0.21	0.57	0.97

### 3.E Dosimetric verification

#### 3.E.1 Point dose measurements

Measured point doses were systematically lower than those predicted by the TPS. For each of the 20 targets from the 10 SRS plans in the study, the average (±1 SD) difference between measured and TPS predicted dose was −1.9% ±1.4%. All of the targets fell within the 5% tolerance set prior to measurement, with the minimum difference measured as −0.1% and the maximum as −4.9%.

There was no trend observed with the point dose results when comparing to target size or number of targets in the plan. However, as seen in Table 6, the difference between the measured and TPS predicted point doses was observed to get larger with increasing distance of the target away from the isocenter of the plan.

**Table 6:** Percentage difference of measured vs TPS predicted point doses as a function of target distance from isocenter

Off axis distance (mm)	Average difference (%)	Standard deviation (%)
0	−0.5	0.3
30	−1.6	1.0
60	−3.2	1.1

#### 3.E.2 Planar dose measurements

**Figure 7:**
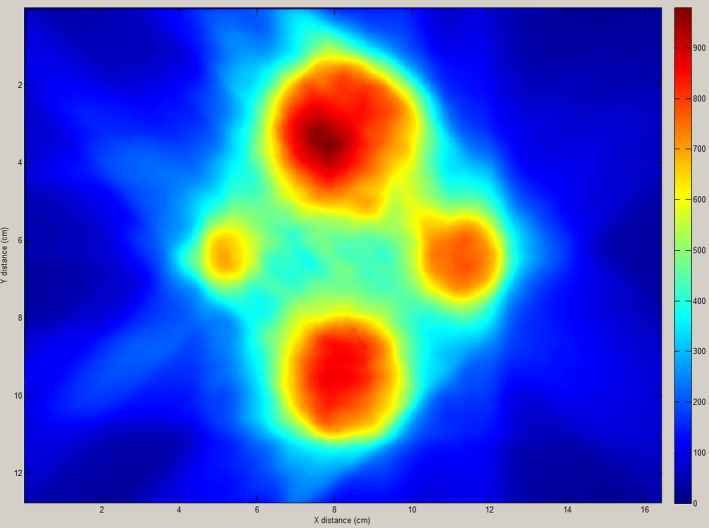
Measured film planar dose for Plan 9. Dose scale is shown in cGy.

For each of the 20 targets from the 10 SRS plans in the study, the average (±1 SD) gamma pass rate for the planar dose verifications was 97.9% ± 1.1%. Each of the targets had a gamma pass rate greater than the 95% tolerance for the 3%/1 mm gamma criteria. The maximum pass rate was 99.4% and the minimum was 95.5%. Figure 7 shows the measured film planar dose for Plan 9.

**Figure 8:**
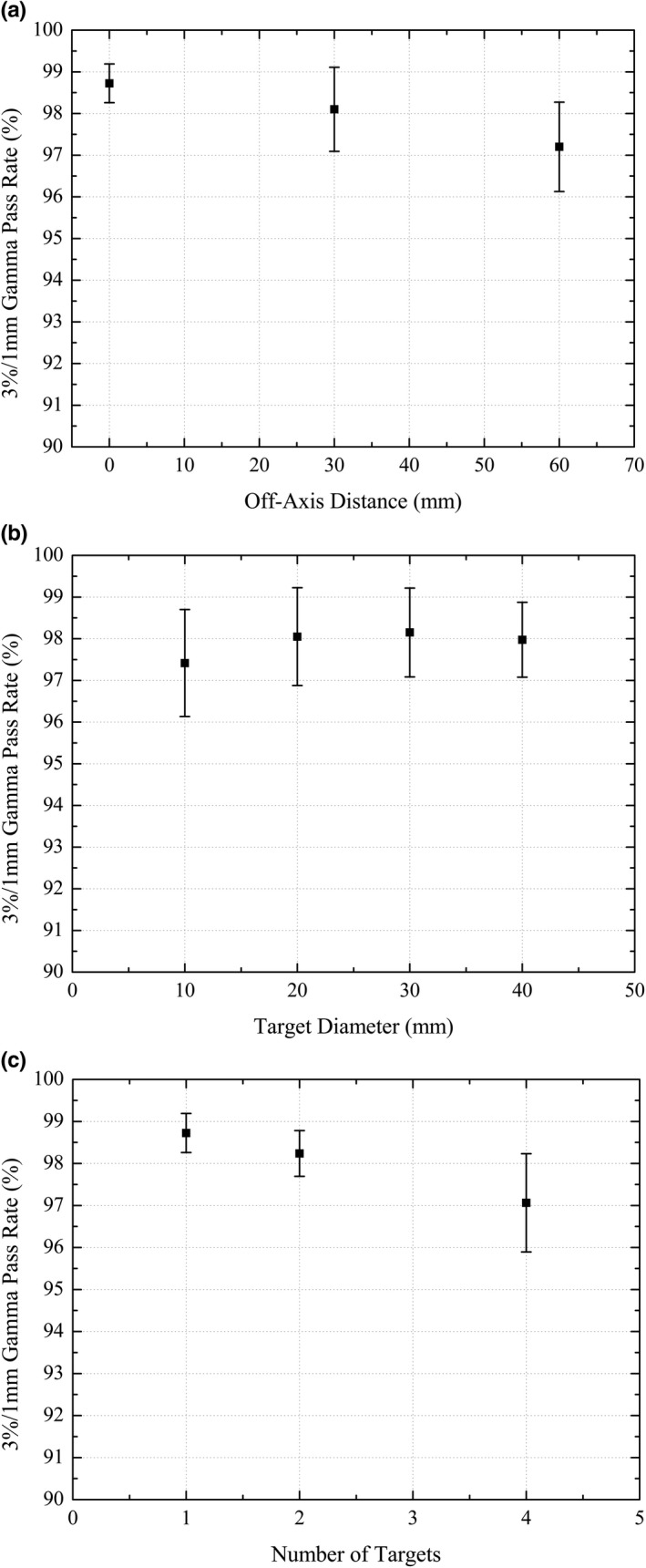
(a) Film gamma pass rate as a function of off‐axis distance, (b) Film gamma pass rate as a function of target size, (c) Film gamma pas rate as a function number of targets in the plan.

Planar dose verification measurements as a function of off‐axis distance, target size, and number of targets in the plan are shown in Figs. 8(a), 8(b), and 8(c), respectively. There was an observable trend of decreasing gamma pass rate with increasing off‐axis distance, decreasing target size and increasing number of targets in the plan.

## 4 DISCUSSION

The analysis of MRI distortions in this study was found to be in agreement with other data from the literature, showing distortions less than 1 mm.^11^, ^25^ Analysis of the CT/MRI registrations errors was also found to be in agreement with the literature, with registration errors being less than 1 mm.^10^, ^26^ Moreover, the results of the CT/MRI registration are within the recommended tolerances from the AAPM Task Group 132 Report^27^ which recommends registration tolerances of half a voxel size, or 0.5 mm in this study.

The methodology utilized in this study to measure the distortion of the MR image requires precise knowledge of the actual distances between the vitamin E capsules in the phantom. This in turn requires precise mechanical tolerances when constructing the inserts. As stated in the Results section, maximum difference between the known and MRI measured distances was comparable to known mechanical tolerance for our phantom. However, it is recommended that if mechanical tolerances less than 0.5 mm cannot be achieved in‐house, then construction of these inserts should be outsourced to a plastic cutting firm, preferably with laser cutting capability.

Analysis of the deviation between CBCT/MV isocenters (or Winston‐Lutz) tests was also in agreement with other studies from the literature. Gao et al.^28^ reported deviations between WLT errors of less than 1 mm up to 3 cm off‐axis, and errors of up to 1.72 mm at an off‐axis distance of 5 cm. However, that study was performed on a Varian 21EX linear accelerator and the WLT pointer was aligned mechanically to the lasers, rather than aligned using the CBCT isocenter as performed in this study. Another study performed by Song, et al.^29^ performed an off‐axis WLT using the same methodology as our study, on a Varian TrueBeam STx linear accelerator up to an off‐axis distance up to 4 cm. The results were similar to those reported here, with maximum off‐center errors of less than 1 mm. As described by Gao et al.,^28^ the increasing deviation between the center of the mechanical and radiation fields with increasing off‐axis distance may be due to the increasing effect of small rotational errors with increasing off‐axis distance. Furthermore, at off‐axis positions, the penumbra of the radiation field becomes asymmetric and the center of the radiation field becomes less well defined, an effect that is magnified with increasing off‐axis distance.

Dosimetric accuracy of the treatment plans in this study was confirmed using both ionization chamber and Gafchromic film measurements. The Gafchromic film measurements confirmed measured vs TPS predicted doses to within 3%/1 mm, and the point dose verification measurements showed agreement with the TPS to within 5%. The measured point doses were observed to measure systematically less than the TPS predicted doses. Similar results using Gafchromic film have previously been reported in the literature for E2E dosimetric verification of SRS plans.^30^, ^31^ Liu, et al.^30^ reported gamma pass rates of greater than 99% for 3%/2 mm gamma criteria, and Dimitriadis, et al.^31^ found an average gamma pass rate of 96.7% for 2%/2 mm gamma criteria. Planar dose verification measurements were observed to have decreasing gamma pass rates with increasing plan complexity.

The E2E phantom presented in this study includes rounding of the end through which the vertex fields enter the phantom. This modification was performed to more closely match the shape of the human head. However, the authors note that such modifications may not be possible for all departments who wish to mimic this study. Rounding of the end of the phantom is not required for VMAT treatments because the beam spends very little time entering through the square corners of the phantom, and good dosimetric accuracy may still be achieved without the rounded end modification. Furthermore, the modifications were performed in the area of the Multi‐Plug insert that does not shadow any of the diodes contained within the ArcCheck phantom (i.e., toward the end of the ArcCheck phantom containing the electronics). This end of the ArcCheck phantom will always be placed at the end of the measured fields, even for merged fields. We have performed VMAT QA with the ArcCheck phantom with both a modified and un‐modified Multi‐Plug insert. Comparison of the two measurements was performed with gamma criteria set to 1%/1 mm. The gamma comparison yielded a pass rate of 100% which we found to be acceptable and concluded that the modification of the Multi‐plug does not affect its use with the ArcCheck.

One limitation of this study is that the contouring uncertainty was not considered in the E2E analysis. Contouring uncertainty should be considered in true E2E validation of a treatment technique as it may contribute significantly toward the total combined geometric accuracy of the technique. Contouring uncertainty of brain metastases has however, been studied previously, with a geometrical uncertainty of approximately 0.3 mm^10^ described.

Another limitation of the study is that intra‐fraction motion was also not considered in the analysis. A study by Wen et al.^32^ was performed with the same linear accelerator and immobilization system used in this study. Intra‐fractional motion was assessed in the study using repeated two‐dimensional kV imaging and optical surface monitoring systems (OSMS). The authors of the study presented excellent agreement between the two systems for the measured intra‐fractional motion among their patient cohort. Average intra‐fraction translations and rotations were measured to be approximately 0.5 mm and 0.5°, respectively. Data obtained from the first five patients treated with the SRS technique at our institution has resulted in similar intra‐fraction translations and rotations to those quoted in the study.

Finally, only up to 4 target volumes were considered in any one plan. This limitation is one that has been implemented locally in our department, based on published randomized clinical trial data.^5^, ^6^, ^7^ However, the authors are aware that it is common for departments to routinely treat more than 4 targets using MTSI plans and we believe that the technique presented in this paper can be easily extended to provide E2E testing for MTSI plans with more than 4 targets. Extension of the E2E technique to MTSI plans with more than 4 targets will be investigated in future studies.

## 5 CONCLUSION

With the growing role of linear accelerators in treating brain metastases patients with SRS, the number of radiation oncology departments around the world providing an SRS service is likely to increase significantly. We have developed a novel and cost‐effective solution for comprehensive E2E testing of these treatments that can incorporate multiple detector types and is easily adaptable to the specific workflows of these departments. The E2E test revealed that targets, even when located in non‐isocentric positions, can consistently be located to within 1 mm using CBCT.

## CONFLICT OF INTEREST

The authors have no conflicts of interest to report.
